# Approaches to tracing the geographic origin of wildlife trade

**DOI:** 10.1093/nsr/nwae286

**Published:** 2024-08-21

**Authors:** Tong Tong Gu, Hao Zhang, Yu Xin He, Jing Yang Hu, Li Yu

**Affiliations:** State Key Laboratory for Conservation and Utilization of Bio-Resource in Yunnan, School of Life Sciences, Yunnan University, China; State Key Laboratory for Conservation and Utilization of Bio-Resource in Yunnan, School of Life Sciences, Yunnan University, China; Southwest United Graduate School, China; State Key Laboratory for Conservation and Utilization of Bio-Resource in Yunnan, School of Life Sciences, Yunnan University, China; State Key Laboratory for Conservation and Utilization of Bio-Resource in Yunnan, School of Life Sciences, Yunnan University, China; State Key Laboratory for Conservation and Utilization of Bio-Resource in Yunnan, School of Life Sciences, Yunnan University, China; Southwest United Graduate School, China

Trade in wildlife is the third largest global illegitimate business after arms and drugs [1]. It presents a significant threat to biodiversity, increasing the risk of species extinctions [2]. Tracing the origins of illegal wildlife trafficking has become a conservation priority. An increasing number of various technologies has been developed to identify illegally traded species and determine the origin of confiscated wild animals and their products, thereby assisting law enforcement agencies in tracing the sources of the trafficking. Here, we summarize the current analytical approaches used to trace the geographical origin of species, including trade record, elemental signature, and molecular genetics analyses (Fig. 1). The principles behind these methods, together with their advantages and disadvantages, and examples of their application regarding geographical traceability, are provided (Fig. 1 and Table S1). We provided new insights into tracing the sources of illegal wildlife trafficking, helping its trade prevention and aiding biodiversity conservation.

**Figure 1.**
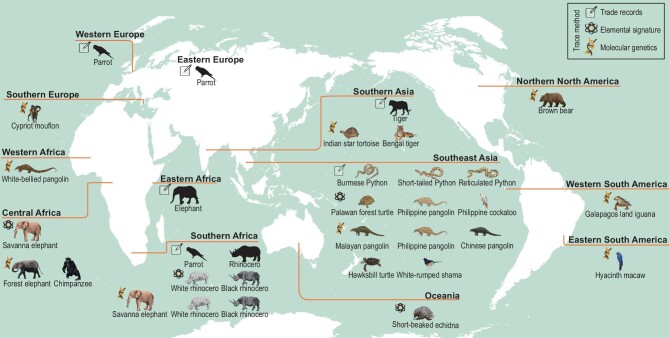
Approaches and applications of the methods currently used to trace the geographic origin of illegally traded wildlife (see the online Supplementary Data for a detailed description). There are two types of animal images shown in the figure. The normal animal illustrations represent examples that can be traced to the specific species. The black animal outline sketch [Elephant, Tiger, and Parrot] represents examples tracing back to a certain animal group but not the specific species. Review drawing number: GS 京(2024)1677号.

## TRACING ILLEGAL WILDLIFE TRAFFICKING USING TRADE RECORDS

Illegal wildlife trading is typically recorded in databases including the Convention on International Trade in Endangered Species of Wild Fauna and Flora (CITES), Wildlife Trade Monitoring Network (TRAFFIC), HealthMap Wildlife Trade (HWT), Global Live Vertebrate Trade Database (GLVTD), and Elephant Trade Information System (ETIS). These records provide illegal trade-related information that includes the seizure year, the wildlife or product quantity and weight, and the trade source and destination, which can be used to help determine the trafficking patterns and trade hotspots. For example, Milliken *et al.* (2013) tracked the trade routes of large-scale (>500 kg) ivory seizures based on trade information in the ETIS, and found that most illegal ivory trade flows shifted from Tanzania to Kenya and Mozambique. Li *et al.* (2023) used GLVTD to perform a network analysis of the global flows of 6664 traded alien vertebrates to quantify their potential invasion risk on local ecosystems, highlighting south and east Asia, Africa, South America and North America as the main export regions for alien vertebrates [3]. Patel *et al.* (2015) analyzed 505 international shipments from HWT reports between 2010 and 2013, and identified key exporters in the illegal trade network of the most frequently recorded species, elephants, rhinoceros and tigers, as Kenya and Tanzania, South Africa, and India, respectively. Based on a TRAFFIC report on the python skins trade in Southeast Asia, Kasterine (2012) highlighted Indonesia and Malaysia as primary sources. Using a large dataset derived from CITES records between 1975 and 2016, Chan *et al.* (2021) found that the major suppliers of the parrot trade had shifted from the Americas, western Europe and Africa, to South Africa, eastern and western Europe.

Trade record analyses offer valuable insights into global trafficking patterns; however, the information is often deficient or incomplete. In addition, even some existing wildlife trade monitoring networks collected tons of information but could not effectively trace the wildlife trafficking, thus entailing the introduction of new tracing methods. On the other hand, the lack of traded species information in the records also poses a challenge regarding the accuracy and comprehensiveness of traceability, and moreover, hampers the ability to discern and monitor those species subjected to illicit trade, as well as hinders the formulation of targeted conservation strategies. Therefore, there is an information gap between trade species information and trade records that needs to be filled, in order to fully assess the impact of trafficking on specific endangered species and ecosystems.

## TRACING ILLEGAL WILDLIFE TRAFFICKING USING ELEMENTAL SIGNATURES

As a result of diet, elemental signatures, unique combinations of different stable isotope and natural element concentrations/abundances, exist in various parts of an animal, including bones, teeth, hair, claws, skin, nails, horns and feathers. These signatures can provide insight into local food web, climate and other environmental parameters over time [4]. They can also be used to determine the source of animals found within the illegal wildlife trade.

Stable isotope analysis (SIA) measures the ratio of different isotopes within a sample and compares them with the natural abundance of the element. It requires expensive equipment and complex sample preparation processes (including grinding and gasification). Van der Merwe *et al.* (1990) and Vogel *et al.* (1990) conducted stable isotope analyses of δ^13^C, δ^15^N and δ^87^Sr in ivory and bones from different regions of Africa, revealing significant differences between African elephant populations, and distinct distribution areas. They proposed that isotope analysis could be used to trace the origin of illegally traded ivory [5,6]. Ishibashi *et al.* (1999) used δ^13^C and δ^15^N ratios and compared 163 ivory samples from 11 range states of savanna elephants (*Loxodonta africana*), tracing them to the Congo, Gabon, and Zaire. Ziegler *et al.* (2016) analyzed more elements (δ^13^C, δ^15^N, δ^18^O, δ^2^H and δ^34^S) from pulverized ivory powder to assign 487 samples from 28 African elephant range states based on reference samples of savanna elephants from 208 sites. Fifty percent of samples could be assigned to within 381 km of their place of origin, and 75% within 1154 km [7]. Besides ivory, stable isotopes have been used to determine the geographic origin of rhinoceros horn. Amin *et al.* (2003) used δ^13^C and δ^15^N to trace seized black rhinoceros (*Diceros bicornis*) samples back to Namibia, Zimbabwe and South Africa, and white rhinoceros (*Ceratotherium simum*) samples to Namibia, Swaziland and South Africa.

The recently developed X-ray fluorescence (XRF) technique can investigate the concentration or abundance characteristics of up to 40 different natural elements. This element signature information can be obtained in real-time, even in the wild, by directly examining the sample with a portable device, and has the advantages of being non-invasive, cost-effective, high-resolution and highly efficient. Brandis *et al.* (2018) measured 24 elements to identify captive-bred and wild-caught short-beaked echidnas (*Tachyglossus aculeatus*) in Australia, and compared the results with corresponding stable isotope data (δ^13^C and δ^15^N). They found that XRF analyses reduced the overall identification error rate to below 4%, compared with SIA. Brandis *et al.* (2023) compared 42 elements and stable isotope data (δ^13^C and δ^15^N) to determine the captive or wild status of three critically endangered, and frequently illegally traded, Philippine species (Palawan forest turtle *Siebenrockiella leytensis*, Philippine cockatoo *Cacatua haematuropygia* and Philippine pangolin *Manis culionensisis*). Again, they found a more accurate assignment was achieved using XRF data than stable isotope data. These studies indicate that XRF provides a valuable forensic tool that can be used to trace the origin of illegally traded wildlife.

Compared with trade record analyses, stable isotope and XRF analyses provide more accurate and quantifiable methods for determining the geographic traceability of traded wildlife. However, the multiple tissue measurements used for elemental signature analyses can produce complex results, as each tissue type may exhibit a distinct elemental composition based on factors such as physiology, diet and environmental exposure. This complexity raises questions regarding the accuracy and applicability of the methods. Future research should focus on refining methodologies, standardizing sampling protocols, and establishing robust calibration frameworks to enhance the reliability and broader applicability of elemental signature analyses in the context of tracing the origin of traded wildlife.

## TRACING ILLEGAL WILDLIFE TRAFFICKING USING MOLECULAR GENETICS (DNA)

With the development of molecular biology, molecular genetics, mainly DNA analysis, has been applied to determine the geographic origins of illegally traded wildlife, by comparing DNA sequences of traded samples with those of voucher specimens of known geographic origin [8].

Mitochondrial (mt) DNA is a small circular molecule comprising 37 genes, with abundant copy numbers, a relatively high mutation rate and a large number of available reference sequences. Gentile *et al.* (2013) used *Cytb* to determine that four confiscated iguana were Galapagos Land Iguana (*Conolophus subcristatus*), which is native to the Galápagos islands of Baltimore and North Seymour. Luczon *et al.* (2016) employed *COI* to identify confiscated pangolins as Philippine pangolins (*Manis culionensisis*), endemic to Palawan. Barbanera *et al.* (2012) used *Cytb* to show that samples from the poached animals had the same haplotype as the wild Cypriot mouflon (*Ovis orientalis ophion*), thus identifying them as Cypriot.

Studies based on mitochondrial phylogenetic geography can also assign seized individuals to specific genetic lineages, and this information can then be used to trace their geographical origins based on the natural distribution of their genetic lineages [9]. For example, Gaubert *et al.* (2016) used *Cytb* to assign the white-bellied pangolin (*Phataginus tricuspis*) into six lineages. By comparing the genetic clustering of the seized samples with reference samples from the six known lineages, the western central Africa lineage has been identified to be the main target of poaching, indicating the prevalence of local illegal trade, while Cameroon and Nigeria play a significant role in the intercontinental illegal pangolin trade. Kennedy *et al.* (2018) used *COI* analyses to demonstrate that the bear paw bones from Market Street Chinatown, San Francisco, USA, had the same base mutation as brown bear (*Ursus arctos*) populations from southwestern Alberta and southeastern British Columbia, Canada, indicating that bear products had been traded from the Pacific Northwest to the San Francisco Bay area. Likewise, LaCasella *et al.* (2021) revealed that 9 of 13 hawksbill turtle (*Eretmochelys imbricata*) products confiscated from Papua New Guinea and the Solomon Islands had the same *D-loop* haplotypes as those from the Solomon Islands rookery.

Microsatellites (STRs) are short sequences, 1–6 nucleotides in length, that are commonly used as hypervariable markers for population and individual assignment, thus enhancing the effectiveness of traceability analyses [10]. Wasser *et al.* (2004) combined genetic and statistical methods to estimate geographic-specific allele frequencies over the entire African elephants’ range based on 16 STR loci, using 315 tissue and 84 scat samples from forest and savanna elephants from 28 locations [11], and infer the geographic origin of 50% of the individual DNA samples to within 500 km of their source, and 80% to within 932 km. Wasser *et al.* (2007) extended this method to analyze the samples that could have one or more common origin(s) simultaneously. This joint analysis performs better than sample-by-sample methods, and was used to infer the geographic origin of the largest ivory seizure since the 1989 ivory trade ban. They revealed that an entire consignment of seized ivory was from savanna elephants, most probably originating from a narrow east-to-west band of southern Africa, centered on Zambia, enabling law enforcement to focus their investigation on a confined area and fewer trade routes. Wasser *et al.* (2008) used the same strategy to examine the geographic origin(s) of two consignments in 2002 involving large volumes of elephant ivory, identifying the source of the confiscated savanna elephant ivory was centered on Zambia, while the forest elephant was centered on Gabon, indicating that criminal groups were engaging in intense exploitation of specific populations. Wasser *et al.* (2015) also traced African elephant ivory from 28 large seizures across Africa and Asia from 1996 to 2014 to two major poaching hotspots in Africa: Gabon and Tanzania. Using the same method to investigate more recent large ivory seizures, from 2016 to 2018, Wasser and Gobush (2019) found that the poaching hotspot of savannah elephants had moved to the Kavango-Zambezi Transfrontier Conservation Area, spanning Botswana, Zimbabwe, Namibia, Zambia and Angola. These efforts again enabled law enforcement to focus their investigation on more specific areas, and provided guidance for law enforcement personnel to refine their scope and target criminal activities more accurately.

Genetic assignment tests based on STR loci have been employed to trace the origins of white and black rhinoceros, iconic African species that are seriously threatened by illegal trade. Harper *et al.* (2018) used 23 STR loci from 3968 rhinoceros individuals, and, based on information from more than 120 criminal cases, to develop the RhODIS^®^ (Rhinoceros DNA Index System) program. This program carries out forensic matching of confiscated African rhinoceros to individuals poached locally, by calculating the allele frequencies of all polymorphic unlinked loci. They traced white rhinoceros from criminal cases to South Africa, and black rhinoceros to South Africa, Kenya and Namibia. Similarly, to combat the illegal trade of the wild Bengal tiger (*Panthera tigris tigris*) in Nepal, Karmacharya *et al.* (2018) generated a genetic database of wild tiger profiles, including different geo-location populations based on eight STRs, revealing Bardia National Park as a poaching hotspot for the Bengal tiger.

Because mtDNA and STR loci represent different genetic backgrounds and reflect maternal and parental genetic sources, respectively, integrating both genetic markers to trace the trade in wildlife can provide more comprehensive genetic information. Gaur *et al.* (2005) combined analyses of mtDNA (*Cytb* and *D-loop*) and six STR loci to identify the origin of rescued star tortoises (*Geochelone elegans*), with most of the rescued individuals having genotypes similar to reference individuals from south India. Similarly, Ghobrial *et al.* (2010), by comparing the *HVRI* sequences and ten STR genotypes of geo-referenced chimpanzee (*Pan troglodytes*) samples from ten locations spanning Cameroon and Nigeria, estimated the geographic origins of 46 rescued chimpanzees to be multiple regions of the Limbe Wildlife Centre in Cameroon and forested areas straddling the Cameroon–Nigeria border. Presti *et al.* (2015) characterized the population genetic structure of the hyacinth macaw (*Anodorhynchus hyacinthinus*) based on ten microsatellites from 98 individuals and 2123 bp of the mtDNA sequence (*ND5, Cytb* and *ND2*) from 80 individuals. They assigned 24 confiscated individuals for sale at the Bolivian border to three geographic regions of Brazil. These examples illustrate how molecular genetics can assist in tracing the origins of species from illegal wildlife trade and, consequently, contribute to more effective actions being taken against such trade.

Advances in genomic sequencing technology have significantly increased the number of loci that can be used in geographic traceability analyses. Genome-wide single nucleotide polymorphisms (SNPs) loci are used because of their distribution, abundance and low genotyping error rate for high-throughput analyses. For instance, based on genome-wide markers across 60 white-rumped shama (*Copsychus malabaricus*) samples from Southeast Asia (Ng *et al.* 2017), genomic assignment demonstrated that all queried Singaporean individuals could be assigned to Sudanic areas outside Singapore, to peninsular Malaysia, adjacent stretches of Sumatra or the sea in between. Several genome-wide analyses have been performed to trace the most trafficked and endangered pangolins. Nash *et al.* (2018) used double-digest restriction site-associated DNA sequencing (ddRADseq) to assign Malayan pangolin (*Manis javanica*) seizures from an illegal trade of unknown origin in Southeast Asia to Borneo, Java and Singapore/Sumatra. Hu *et al.* (2020), based on the population genomic data analyses, speculated that confiscated Malayan pangolins in Yunnan, China, mainly originated from mainland and Southeast Asia islands (except for Java), while confiscated Chinese pangolins (*Manis pentadactyla*) in Yunnan mainly originated from Southern China, Myanmar and Thailand. Tinsman *et al.* (2023) used a genomic approach to reveal five distinct populations of the white-bellied pangolin by analyzing 111 samples collected from known geographic localities in Africa. They then sampled 643 scales confiscated from Asia between 2012 and 2018, and highlighted that poaching pressures have moved from west to central Africa, with Cameroon's southern border recently emerging as a site of intense poaching. Thus, based on the increased number of loci, genomic analyses enable monitoring of changes in poaching patterns in near real-time, and offer more opportunities for tracing the origin of illegally traded wildlife, enabling more targeted and more effective anti-poaching measures.

The continuous updating of genetic markers combined with known specimens from more geographical sources represents a substantial advancement in tracing the origins of trafficked wildlife, providing vital insights that can be used to combat the illegal trade of multiple species across many regions. However, when genetic markers are absent or biological materials are degraded, such as in cases involving heavily processed wildlife products, DNA extraction may be impractical or insufficient, rendering genomic methods ineffective. Moreover, the accuracy and reliability of traceability based on DNA analyses heavily depends on the availability of samples with known geographic origins, of which the number is still limited.

## CONCLUSIONS AND PROSPECTS

The technologies used to trace the geographic origins of illegally traded wildlife are vital for efficient monitoring and curtailment of trafficking. This paper provides an overview of the research status and challenges of the major approaches currently in use, highlighting their application and substantial potential in tracing traded wildlife and combating illegal trade.

Combining multiple and complementary approaches to improve the traceability of the wildlife trade is essential. In addition, innovative approaches are needed to help identify the origins of illegal trade. For example, global positioning system (GPS) data has been used successfully in tracking trade in egg-laying reptiles and birds [12]; intelligent data analysis and deep learning have also accelerated the identification and tracking of traded origins in rhinoceros. For example, Amin *et al.* (2003) has used intelligent analysis to track the origin of illegally traded and confiscated African rhinoceros horns based on isotope data. Through large-scale data analysis testing, feature extraction, and pattern recognition, intelligent data analysis and deep learning not only accelerate the identification and tracking of species sources, providing opportunities for automated analysis, but more importantly, they are not assumptions or data-driven, can ‘learn’ from past data, and predict future trends in wildlife trade with higher accuracy than before [13]. We expect that as data on the illegal trade of species of concern becomes increasingly more comprehensive, this method will demonstrate its application prospects.

Establishing the species-specific database and multi-dimensional data, relative to the current broad-spectrum database of multiple species, is conducive for precise tracing of illegal trade and targeted crime prevention. Currently, the available databases focus primarily on import/export country information and seizure quantities, and lack traded species-specific information, thus hindering the ability to identify and monitor the information of species involved in illegal trade. In addition, integrating the comprehensive geographic, phenotypic, elemental signature and genetic data to establish a multi-dimensional database would also enhance tracking capabilities by providing the detailed information necessary for accurate identification and monitoring of trafficked species. To achieve this, experts on taxonomy, genetics, bioinformatics, and information technology are needed.

Monitoring the trade of species that are not listed as threatened or endangered should be also considered [1], in order to prevent them from becoming endangered as a result of trade trafficking. In addition, the trade has been shown to play an important role in the facilitation of disease transmission, and zoonotic diseases may be transmitted regardless of the conservation status of the species [14]. Therefore, linking the origin of illegally traded species with the transmission pathways of zoonotic diseases can identify potential hotspots of zoonotic diseases and help prevent their spread.

Collecting and obtaining data of the reference samples from known geographic localities is key to tracing the origins of illegally traded wildlife [15]. Without such detailed reference data, tracing the origins of illegal wildlife trafficking will become challenging, hindering law enforcement agencies’ ability to effectively combat this trade. Thus, it is imperative to prioritize the collection and utilization of reference samples in wildlife trade monitoring and enforcement strategies.

The illegal trade in wildlife is contributing to the global loss of biodiversity, and tracing the geographical origins of illegally traded species and products is vital in order to combat trafficking and help conservation efforts. Therefore, a comprehensive global action program, including reducing consumer demand, strengthening law enforcement measures, enhancing international research cooperation, and promoting sustainable alternatives to wildlife products [16], is necessary for preventing the illegal wildlife trade and for biodiversity conservation.

**NOTE:** Due to space limitations, all citations to the application examples based on these approaches described in the text but not cited in the text are provided in the Supplementary material.

## Supplementary Material

nwae286_Supplemental_File
