# Supplementary material for: Approaches to tracing the geographic origin of wildlife trade
Source: Natl Sci Rev. 2024 Aug 21;11(9):nwae286. doi: 10.1093/nsr/nwae286 (PMC11441321; doi:10.1093/nsr/nwae286)
Supplement: nwae286_Supplemental_File [file nwae286_supplemental_file.docx]

**Supplementary Material for “Approaches to tracing the geographic origin of wildlife trade”**

Tong Tong Gu^1^, Hao Zhang^1,2^, Yu Xin He^1^, Jing Yang Hu^1,^ * and Li Yu^1,2,^*

^1^State Key Laboratory for Conservation and Utilization of Bio-Resource in Yunnan, School of Life Sciences, Yunnan University, Kunming, China

^2^Southwest United Graduate School, Kunming, China

**Note:** Due to space limitations, all citations to the method application examples based on these approaches described in the text but not cited in the text are provided in the Supplementary material.

**Supplementary Table S1**. Approaches and applications of the methods currently used to trace the geographic origin of illegally traded wildlife. The reference number corresponds to the numbers behinds the species names in Figure 1.

| **Approach** | **Common name** | **Latin name** | **Traceability results** | **Authors** | **Year** | **Reference number** |
| --- | --- | --- | --- | --- | --- | --- |
| **Trade records analysis** | Elephant | / | Kenya and Tanzania | Patel et al. | 2015 | [17] |
|  | Rhinocero |  | South Africa |  |  |  |
|  | Tiger |  | India |  |  |  |
|  | Burmese Python | *Python molurus bivittatus* | Indonesia and Malaysia | Kasterine et al. | 2012 | [18] |
|  | Reticulated Python | *Python reticulatus* |  |  |  |  |
|  | Short-tailed Pythons | *Python curtus*,  *P. brongersmai* *P. breitensteini* |  |  |  |  |
|  | Parrot | / | South Africa, eastern and western Europe | Chan et al. | 2021 | [19] |
| **Elemental signatures** | Savanna elephant | *Loxodonta africana* | Congo, Gabon and Zaire | Ishibashi | 1999 | [20] |
|  | White rhinocero | *Ceratotherium simum* | Namibia, Swaziland and South Africa | Amin et al. | 2003 | [21] |
|  | Black rhinocero | *Diceros bicornis* | Namibia, Zimbabwe and South Africa |  |  |  |
|  | Short-beaked echidna | *Tachyglossus aculeatus* | Australia | Brandis et al. | 2018 | [22] |
|  | Philippine cockatoo | *Cacatua haematuropygia* | Philippine | Brandis et al. | 2023 | [23] |
|  | Palawan forest turtle | *Siebenrockiella leytensis* |  |  |  |  |
|  | Philippine pangolin | *Manis culionensisis* |  |  |  |  |
| **Molecular analyses (DNA and genomic analyses)** | Galapagos land iguana | *Conolophus subcristatus* | Gala´pagos islands | Gentile et al. | 2013 | [24] |
|  | Philippine pangolin | *Manis culionensisis* | Philippine | Luczon et al. | 2016 | [25] |
|  | Cypriot mouflon | *Ovis orientalis ophion* | Cyprus | Barbanera et al. | 2012 | [26] |
|  | White-bellied pangolin | *Phataginus tricuspis* | Gabon (northwestern Gabon), central Africa (Central African Republic, Democratic Republic of Congo, northwestern Tanzania), western central Africa (Cameroon, Equatorial Guinea, northeastern Gabon), the Dahomey Gap (Togo, Benin, western Nigeria), western Africa (Liberia, Guinea, Ivory Coast) and Ghana (Ghana) | Gaubert et al. | 2016 | [27] |
|  | White-bellied pangolin | *Phataginus tricuspis* | western central Africa | Zanvo et al. | 2022 | [28] |
|  |  |  |  | Dipita et al. | 2023 | [29] |
|  | White-bellied pangolin | *Phataginus tricuspis* | Cameroon (western central Africa lineage) and Nigeria (Dahomey Gap lineage) | Zhang et al. | 2020 | [30] |
|  | Brown bear | *Ursus arctos* | southwestern Alberta and southeastern British Columbia, Canada | Kennedy et al. | 2018 | [31] |
|  | Hawksbill turtle | *Eretmochelys imbricata* | Solomon Islands | LaCasella et al. | 2021 | [32] |
|  | Savanna elephant | *Loxodonta africana* | A narrow east-to-west band of southern Africa, centered on Zambia | Wasser et al. | 2007 | [33] |
|  | Forest elephant | *Loxodonta cyclotis* | Centered on Gabon | Wasser et al. | 2008 | [34] |
|  | Savanna elephant | *Loxodonta africana* | Centered on Zambia |  |  |  |
|  | Forest elephant | *Loxodonta cyclotis* | Centered on Gabon | Wasser et al. | 2015 | [35] |
|  | Savanna elephant | *Loxodonta africana* | Centered on Tanzania |  |  |  |
|  | Savanna elephant | *Loxodonta africana* | Kavango-Zambezi Transfrontier Conservation Area, spanning Botswana, Zimbabwe, Namibia, Zambia and Angola | Wasser and Gobush | 2019 | [36] |
|  | White rhinocero | *Ceratotherium simum* | South Africa | Harper et al. | 2018 | [37] |
|  | Black rhinocero | *Diceros bicornis* | South Africa, Kenya and Namibia |  |  |  |
|  | Bengal tiger | *Panthera tigris tigris* | Bardia National Park in Nepal | Karmacharya et al. | 2018 | [38] |
|  | Indian star tortoise | *Geochelone elegans* | South India | Gaur et al. | 2005 | [39] |
|  | Chimpanzee | *Pan troglodytes* | multiple regions of the Limbe Wildlife Centre in Cameroon and forested areas straddling the Cameroon-Nigeria border | Ghobrial et al. | 2010 | [40] |
|  | Hyacinth macaw | *Anodorhynchus hyacinthinus* | Northeastern Brazil | Presti et al. | 2015 | [41] |
|  | White-rumped shama | *Copsychus malabaricus* | Sudanic areas outside of Singapore, either peninsular Malaysia, adjacent stretches of Sumatra or the sea in between | Ng et al. | 2017 | [42] |
|  | Malayan pangolin | *Manis javanica* | Borneo, Java and Singapore/Sumatra | Nash et al. | 2018 | [43] |
|  | Malayan pangolin | *Manis javanica* | mainland and Southeast Asia islands (except for Java island) | Hu et al. | 2020 | [44] |
|  | Chinese pangolin | *Manis pentadactyla* | Southern China, and Myanmar and Thailand of Southeast Asia |  |  |  |
|  | White-bellied pangolin | *Phataginus tricuspis* | central Africa | Tinsman et al. | 2023 | [45] |

**REFERENCES**

17. Patel NG, Rorres C, Joly DO. Quantitative methods of identifying the key nodes in the illegal wildlife trade network. *Proc Natl Acad Sci USA* 2015; **112**: 7948-53.

18. Kasterine A. The trade in southeast Asian python skins. *ITC* 2012.

19. Chan DTC, Poon ESK, Wong ATC *et al.* Global trade in parrots - Influential factors of trade and implications for conservation. *Glob Ecol Conserv* 2021; **30**: e01784.

20. Ishibashi H, Takeuchi T, Whyte I *et al.* δ^15N and δ^13C measurements from the African elephant, *Loxodonta africana*, used for ivory sourcing. *Bullitin of the Graduate School of Social and Cultural Studies, Kyushu University* 1999; **5**: 1-8.

21. Amin R, Bramer M, Emslie R. Intelligent data analysis for conservation: experiments with rhino horn fingerprint identification. *Knowl-Based Syst* 2003; **16**: 329-36.

22. Brandis KJ, Meagher PJB, Tong LJ *et al.* Novel detection of provenance in the illegal wildlife trade using elemental data. *Sci Rep* 2018; **8**: 15380.

23. Brandis KJ, Meagher P, Schoppe S *et al.* Determining the provenance of traded wildlife in the philippines. *Animals (Basel)*. 2023; **13**: 1-14.

24. Gentile G, Ciambotta M, Tapia W. Illegal wildlife trade in Galápagos: molecular tools help the taxonomic identification of confiscated iguanas and guide their rapid repatriation. *Conserv Genet Resour* 2013; **5**: 867-72.

25. Luczon AU, Ong PS, Quilang JP *et al.* Determining species identity from confiscated pangolin remains using DNA barcoding. *Mitochondrial DNA B* 2016; **1**: 763-6.

26. Barbanera F, Guerrini M, Beccani C *et al.* Conservation of endemic and threatened wildlife: molecular forensic DNA against poaching of the Cypriot mouflon (*Ovis orientalis ophion*, Bovidae). *Forensic Sci Int Gen* 2012; **6**: 671-5.

27. Gaubert P, Njiokou F, Ngua G *et al.* Phylogeography of the heavily poached African common pangolin (Pholidota, *Manis tricuspis*) reveals six cryptic lineages as traceable signatures of Pleistocene diversification. *Mol Ecol* 2016; **25**: 5975-93.

28. Zanvo S, Djagoun C, Azihou AF *et al.* Can DNA help trace the local trade of pangolins? Conservation genetics of white-bellied pangolins from the Dahomey Gap (West Africa). *BMC Ecol Evol* 2022; **22**: 16.

29. Dipita AD, Missoup AD, Aguillon S *et al.* Genetic tracing of the white-bellied pangolins (Phataginus tricuspis) trade in western central Africa. *bioRxiv* 2023.

30. Zhang HR, Ades G, Miller MP *et al.* Genetic identification of African pangolins and their origin in illegal trade. *Global Ecology and Conservation* 2020; **23**: e01119.

31. Kennedy JR, Rogers L, Kaestle FA. Ancient DNA evidence for the regional trade of bear paws by Chinese diaspora communities in 19th-century western North America. *J Archaeol Sci* 2018; **99**: 135-42.

32. LaCasella EL, Jensen MP, Madden Hof CA *et al.* Mitochondrial DNA profiling to combat the illegal trade in tortoiseshell products. *Front Mar Sci* 2021; **7**: 1-10.

33. Wasser SK, Mailand C, Booth R *et al.* Using DNA to track the origin of the largest ivory seizure since the 1989 trade ban. *Proc Natl Acad Sci USA* 2007; **104**: 4228-33.

34. Wasser SK, Joseph Clark W, Drori O *et al.* Combating the illegal trade in African elephant ivory with DNA forensics. *Conserv Biol* 2008; **22**: 1065-71.

35. Wasser SK, Brown L, Mailand C *et al.* Genetic assignment of large seizures of elephant ivory reveals Africa’s major poaching hotspots. *Science* 2015; **349**: 84-7.

36. Wasser SK, Gobush KS. Conservation: monitoring elephant poaching to prevent a population crash. *Curr Biol* 2019; **29**: R627-30.

37. Harper C, Ludwig A, Clarke A *et al.* Robust forensic matching of confiscated horns to individual poached African rhinoceros. *Curr Biol* 2018; **28**: 13-4.

38. Karmacharya D, Sherchan AM, Dulal S *et al.* Species, sex and geo-location identification of seized tiger (*Panthera tigris tigris*) parts in Nepal-A molecular forensic approach. *PLoS One* 2018; **13**: e0201639.

39. Gaur A, Reddy A, Annapoorni S *et al.* The origin of Indian Star tortoises (Geochelone elegans) based on nuclear and mitochondrial DNA analysis: A story of rescue and repatriation. *Conserv Genet* 2005; **7**: 231-40.

40. Ghobrial L, Lankester F, Kiyang JA *et al.* Tracing the origins of rescued chimpanzees reveals widespread chimpanzee hunting in Cameroon. *BMC Ecol* 2010; **10**: 2.

41. Presti FT, Guedes NM, Antas PT *et al.* Population genetic structure in Hyacinth Macaws (*Anodorhynchus hyacinthinus*) and identification of the probable origin of confiscated individuals. *J Hered* 2015; **106**: 491-502.

42. Ng EYX, Garg KM, Low GW *et al.* Conservation genomics identifies impact of trade in a threatened songbird. *Biol Conserv* 2017; **214**: 101-8.

43. Nash HC, Wirdateti, Low GW *et al.* Conservation genomics reveals possible illegal trade routes and admixture across pangolin lineages in Southeast Asia. *Conserv Genet* 2018; **19**: 1083-95.

44. Hu JY, Hao ZQ, Frantz L *et al.* Genomic consequences of population decline in critically endangered pangolins and their demographic histories. *Natl Sci Rev* 2020; **7**: 798-814.

45. Tinsman JC, Gruppi C, Bossu CM *et al.* Genomic analyses reveal poaching hotspots and illegal trade in pangolins from Africa to Asia. *Science* 2023; **382**: 1282-6.
